# Robotic thoracic surgery: lessons learned from the first 1,000 procedures

**DOI:** 10.3389/fsurg.2024.1417787

**Published:** 2024-09-12

**Authors:** Marion Durand, Lee S. Nguyen, Frankie Mbadinga, Maksim Pryshchepau, Hadrien Portefaix, Nouha Chaabane, Stanislas Ropert, Naziha Khen-Dunlop

**Affiliations:** ^1^ Thoracic Surgery Department, Groupe Hospitalier Privé Ambroise Paré Hartmann, Neuilly-Sur-Seine, France; ^2^ Research and Innovation Department, Groupe Hospitalier Privé Ambroise Paré Hartmann, Neuilly-Sur-Seine, France; ^3^ Thoracic Surgery Department, University Hospital Centre, Rouen, France; ^4^ Anesthesiology Department, Groupe Hospitalier Privé Ambroise Paré Hartmann, Neuilly-Sur-Seine, France; ^5^ Oncology Department, Groupe Hospitalier Privé Ambroise Paré Hartmann, Neuilly-Sur-Seine, France; ^6^ Pediatric Surgery Department, Necker-Enfants Maladies University Hospital, Paris, France

**Keywords:** robot-assisted thoracic surgery, RATS, lobectomy, segmentectomy, non-small cell lung cancer, sub-lobar resection

## Abstract

**Introduction:**

The aim of this study was to evaluate the impact of the thoracic robotic approach in a high-volume center regarding procedures and clinical outcomes after 1,000 procedures.

**Methods:**

In a single-center subset of the Epithor® database, a prospective cohort database of French thoracic surgery, we analyzed procedural characteristics and clinical outcomes from February 2014 to April 2023. A surgical technique for lung surgery was conducted with a four-arm closed chest with the port access approach and vascular sewing and knotting were preferred over stapling. Statistical analysis was performed using the Chi-2 test for discontinuous variables and the Mann–Whitney–Wilcoxon test for continuous variables. Tests were considered significant for a *p*-value <0.05.

**Results:**

Robotic thoracic surgery was used in anatomical lung resection in 85% of the cases. Over the study period, 1,067 patients underwent robotic surgery, of which 509 had lobectomies and 391 segmentectomies. In the segmentectomy group vs. lobectomy group we observed a shorter length of stay (9 ± 7 vs. 7 ± 5.6 days, *p* < 0.001), a shorter surgery time (99 ± 24 vs. 116 ± 38 min, *p* < 0.001) a lower conversion rate (*n* = 2 vs. *n* = 17, *p* = 0.004), and a lower complication rate (28% vs. 40%, *p* = 0.009, mainly Clavien–Dindo II, 18% and 28%, respectively). For cancer treatment surgery, we found more previous cancer in the segmentectomy group (48% vs. 26%, *p* < 0.001). We also observed a progressive change of lobectomy vs. segmentectomy from 80%/20% to 30%/70% over the 9 years.

**Discussion:**

A robotic platform is an appropriate tool to perform anatomical lung resection and especially to develop a safe and systematic approach to lung-sparing sub-lobar resection.

## Introduction

Between the 1950s and the end of the last century, discussions about lung cancer treatment revolved around pneumonectomy and lobectomy with node harvest with initial in-hospital mortality averaging 25% (1, 2). Since then, major technical and non-technical improvements have allowed us to reach quality and safety in lung cancer surgical treatment. In the last decade, tremendous evolution in technology has deeply changed the surgical approach, keeping three oncologic dogmas: R0 margin, “en-bloc” anatomical lung resection, and radical node harvest (3).

Robotic surgery, characterized by telemanipulation or immersive surgery, offers optimized visual and technical conditions to perform anatomical lung resection and node harvesting in an immersive and enhanced skills approach. Since its introduction, robotic surgery has been a subject of considerable debate, often compared to previous minimally invasive approaches using video-assisted thoracic surgery. The robotic tool provides technical capabilities akin to bimanual skilled dissection, similar to open surgery. However, unlike the thoracoscopic approach, which has limitations due to the rigidity of instruments and limited vision inside the chest (two-dimensional, limited camera versatility), the robotic approach benefits from enhanced vision (three-dimensional and 10-times magnification) and three hand-wrist instruments.

The technical capabilities offered by the robotic tool extend beyond bimanual skilled dissection in open surgery. Some advantages have been shown, including minimal musculoskeletal sequelae, reduced postoperative pain, shorter hospitalization, and lower 30-day mortality (4–7). This development has been supported by the introduction of new generations of tele-manipulators, featuring smaller and more versatile arms and smaller cameras, enabling more complex surgeries.

Robotic surgery for anatomical lung resection is steadily gaining prominence. However, according to the French prospective database Epithor® between 2010 and 2020, only 7% of anatomical lung resections were performed with the robotic platform (8).

Recent publications emphasize the benefit of the lung-sparing strategy for T1 less than 2 cm N0 cancer through sub-lobar resection (9, 10). Difficulties in controlling parenchymal margins and nodule locations can be optimized with preoperative reconstructions and intersegmental planning (11).

Over the past 20 years, the Epithor® registry has aimed to prospectively include all patients undergoing thoracic surgery in France, serving as a real-life database for collecting preoperative, operative, postoperative, and follow-up data for patients treated for thoracic disease.

This study aims to examine the early outcomes of robotic surgery and the impact of segmentectomy based on data from the French Epithor® database, analyzing the first consecutive 1,067 robotic procedures from a high-volume center and comparing the characteristics and outcomes of the segmentectomy group vs. the lobectomy group.

## Method

### Ethical statement

All the data were extracted from the French national prospective database Epithor® (Epidemiology for Thoracic Surgery), managed by the French National Council of Thoracic and Cardiovascular Surgery (SFCTCV), which is the sole representative of the professional practice of Thoracic and Cardiovascular Surgery in France.

The database was created in 2002 and set up after the specific authorization from the Commission Nationale Informatique et Libertés—CNIL no. 809833 and the French General Data Protection Regulation (Règlement Général sur la Protection des Données—RGPD).

Patient informed consent was obtained to gather the database.

The surgeon contributing to the registry is bound by the rules of professional ethics, and in particular by professional secrecy. He has signed a charter of use and confidentiality.

### Study design

This is a retrospective cohort study, including consecutive patients operated on in a single center from February 2014 to January 2023. Preoperative clinical status, technical aspects, surgical features, and postoperative outcomes were analyzed. For oncologic cases, the characteristics and outcomes of the segmentectomy group vs. the lobectomy group were also compared.

### Surgical approach for lung resection

As previously described, we used the four-arm totally endoscopic “W approach” (12).

The original four-arm da Vinci Surgical System (Intuitive Surgical, California, USA) was used for all procedures. From February 2014 to September 2017, the Si® system was used and from October 2017 to January 2023, it was upgraded to the Xi® system.

For Xi®, the cart was placed in the same position in the room, and the boom was switched as required to ensure alignment of the scapula line with the camera arm. A 30° camera was used.

The patient was placed in a lateral position. The tip of the scapula and the camera position were first marked. The other trocars were then marked, and the correct projection was checked once the camera was in place. A fifth trocar, designated as port access, was always added to let the bed assistant help with suction and exposure, ensure a light capnothorax (pressure 5 mmHg, flow 10 L/min), and also for stapling delegation.

#### Instrument armamentarium

As the standard setting, we prefer as follows: for the right hand we use the permanent cautery spatula (ref 420184) or the suture cut needle driver SutureCut™ (ref 420296), for the left hand we use fenestrated bipolar forceps (ref 420205), and for the assistant arm we use the ProGrasp™ forceps (ref 420093). In cases of special need, we use the Tip-up Fenestrated Grasper (ref 470347), Monopolar Curved Scissors (ref 470179), Maryland Bipolar Forceps (ref 471172), and when needed, a Vessel Sealer Extend (ref 480422).

#### Surgical strategy

We favor the use of hand-wrist dissection for vessel control, ligation, and suture to maintain surgeon hard skills, reproduce open surgery techniques, and optimize the use of the robotic system’s capacity.

The artery ligation usually requires two rows of Silk 0 sutures, cut to 10 cm lengths, and distal electrocoagulation. For vein control, the first ligation was with silk wire size 0, 10 cm length double knotted with braided 22 mm needle 2/0 wire 10 or 15 cm length, with distal electrocoagulation with the spatula. Exceptionally, an automatic stapler could be used for vessel suture with a preference for Echelon™ Powered Stapler (Ethicon Endosurgery, US), 35 mm white load, or da Vinci Sureform™ 45 mm tip up.

#### Intersegmental plan definition

In the case of segmentectomy, for lung mapping, we use indocyanine green as previously described (13). After vessel ligation, we inject a dose of 8 ml IV flash for all patients, which means 20 mg of indocyanine green (ICG), and then flushed with 10 ml saline straight after. The injection should be closest to the patient rather than in the line to ensure a bolus. After approximately 20 s, the marking can be performed after switching on the infrared light of the camera.

### Statistical analyses

Quantitative data were expressed as means and standard deviation.

Categorical data were expressed as numbers and percentages. Data analysis was done using IBM SPSS statistics software (Released 2017; IBM SPSS Statistics for Windows, Version 25.0, IBM Corp., Armonk, NY, USA). Continuous variables were compared using the Mann–Whitney–Wilcoxon test. The frequencies of categorical variables were compared using Fisher's exact test. A *p*-value < 0.05 was considered significant.

## Results

### Global population result

**Figure 1:**
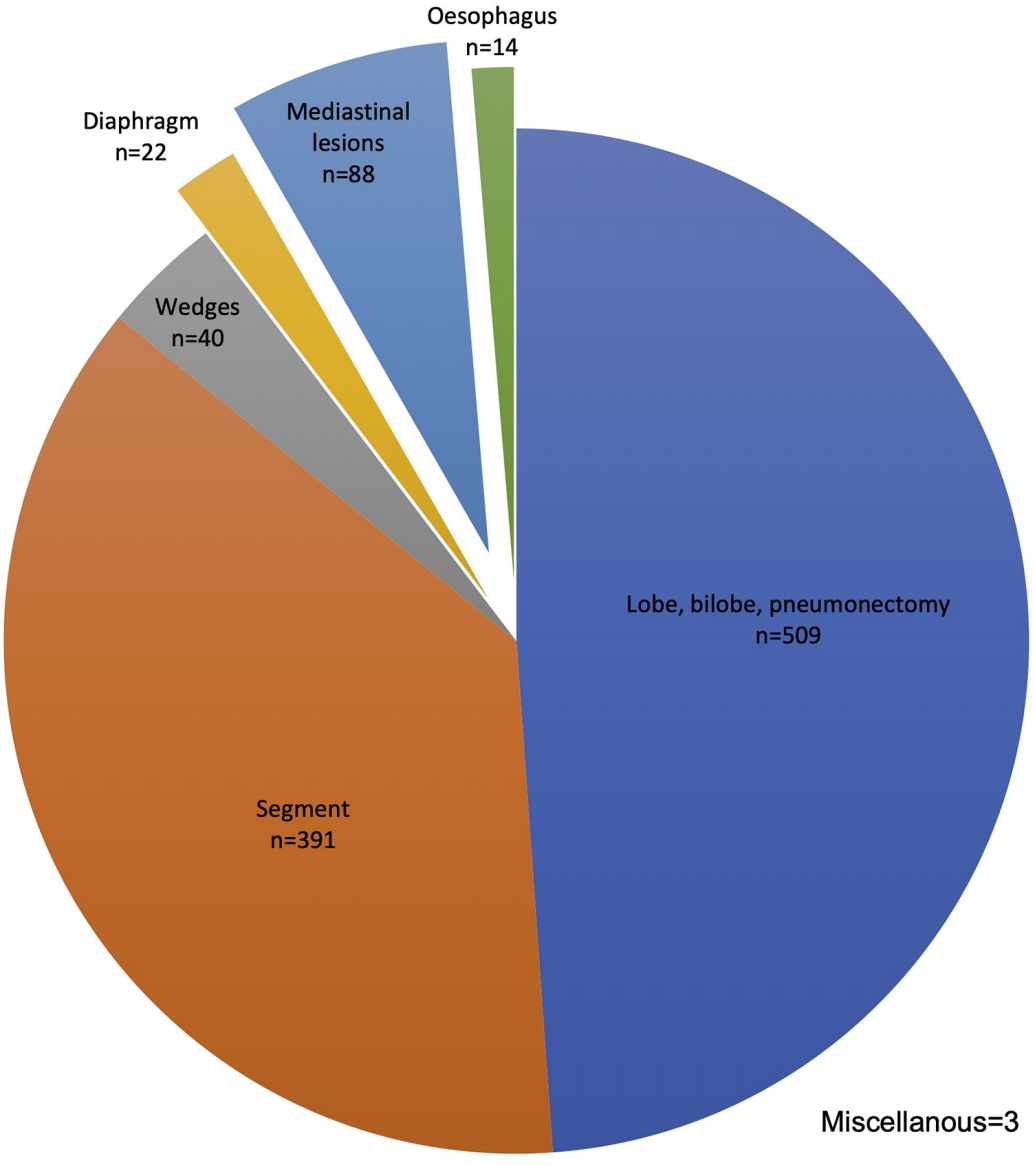
Robotic procedure distribution *n* = 1,067. Upper bilobectomy *n* = 15, lower bilobectomy *n* = 4, and pneumonectomy *N* = 7.

During the study time, 1,067 consecutive patients were operated on using the da Vinci robotic platforms Si® and Xi®. Among these, 940 (88%) underwent lung surgery, mainly major resections: lobe, bi-lobe, or pneumonectomy (509; 48%) and segments (391; 37%). The procedure distribution is given in Figure 1.

**Figure 2:**
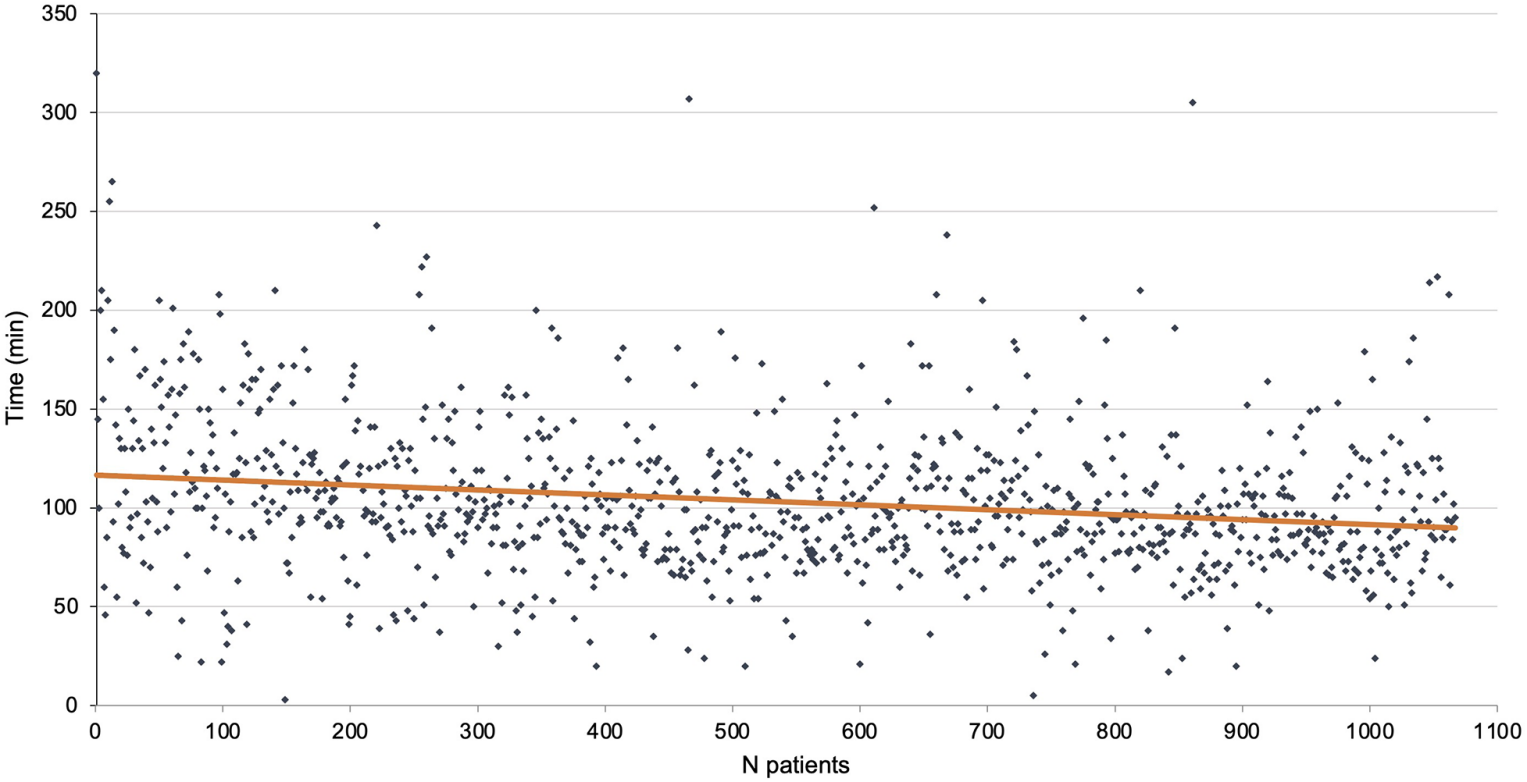
Console time per procedure (orange line shows a linear tendency curve).

The total length of the procedure was 114.9 ± 39.2 min. It was 143.8 ± 53.9 min for the first 100 patients at the beginning of the study period and 107.8 ± 35.3 min for the last 100 patients of the cohort. The mean length of console time was 103.2 ± 37.7 min. There were 30 conversions to thoracotomy (2.8%) of which 7 were emergencies (0.7%). The mean length of stay was 7.8 ± 7.3 days. The console time is reported in Figure 2.

Conversion reasons are reported in Table 1 (*n* = 30). Most were due to bleeding. None ended uncontrolled bleeding and the level of resection was the one initially planned. No deaths in the operating room were reported in this series. Anesthesia issues (*n* = 2) at the beginning of the study period were due to ventilation problems, one of which resulted in cardiac arrest although the patient was successfully resuscitated and the procedure was stopped thereafter; another required conversion as exclusion was impossible. Non-vascular dissection events (*n* = 8) included full pleurodesis, non-dissectible hilum, and bronchial leak post-stapling. Vascular dissection events (*n* = 12) were mainly due to pulmonary invasion requiring pneumonectomy or extended sleeve. Two procedures required conversion due to mediastinal disease and were converted through a sternotomy (Invasion of the innominate venous trunk, mass size over 10 cm). Two robot position issues impairing the procedure were encountered at the beginning of the study period with the Si® system.

**Table 1:** Conversion reasons*.*

	Conversion, *N*	Emergency conversion, *N*
Bleeding	6	6
Anesthesia issue	2	1
Vascular dissection events	12	
Non-vascular dissection events	8	
Robot position	2	

After 338 cases and during the fourth year, the robotic platform was upgraded to a Xi® system. The results of the two periods are given in Table 2. We observed a significant decrease in all the reported parameters from the procedures including timings, complications, conversions, and length of stay.

**Table 2:** Perioperative results comparison between the Si/Xi periods.

	Length of surgery (min)	Console time (min)	Docking time (min)	Conversion, *n* (%)	LOS (days)	Complications, *n* (%)
Si period (338)	150.8 ± 43.2	103.2 ± 36.2	14.2 ± 4.3	17 (5%)	9 ± 7	128 (38%)
Xi period (729)	108.9 ± 34.6	98.3 ± 33.7	10.6 ± 3.3	13 (1.7%)	7 ± 7.5	211 (29%)
*p*	<0.001	<0.001	<0.001	0.003	<0.004	0.04

LOS, length of stay in days.

Time unit is minutes.

**Figure 3:**
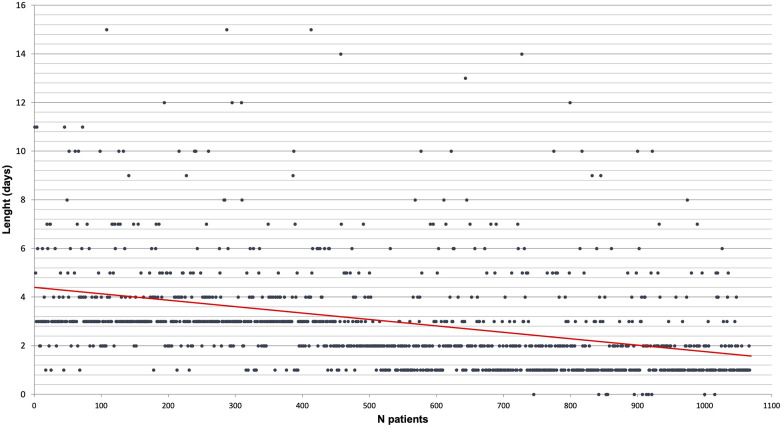
Chest drain length by chronological order of patient (each patient is a dot and the red line is a linear tendency curve).

The length of chest drain duration is reported in Figure 3. For each procedure, only one chest tube was placed at the end of the procedure. During the study period, the strategy improved by lowering the length of chest tube remaining, the device used, and the level of pressure.

### Complications and mortality

There were 339 complications (31.8%) according to the Clavien–Dindo scale (14) and 13 deaths (1.2%). The deaths involved 12 men and 1 woman, with a mean age of 70.1 years old (range 58–84) and mean forced expiratory volume (FEV) 66%, all being smokers.

The deaths occurred after nine lobectomies or bilobectomies, two segmentectomies, one Lewis Santy surgery, and one wedge resection. The postoperative complications were refractory lung infection (seven cases), stroke (three cases), COVID-19 (two cases), and one fistula.

### Anatomical lung resection results

**Figure 4:**
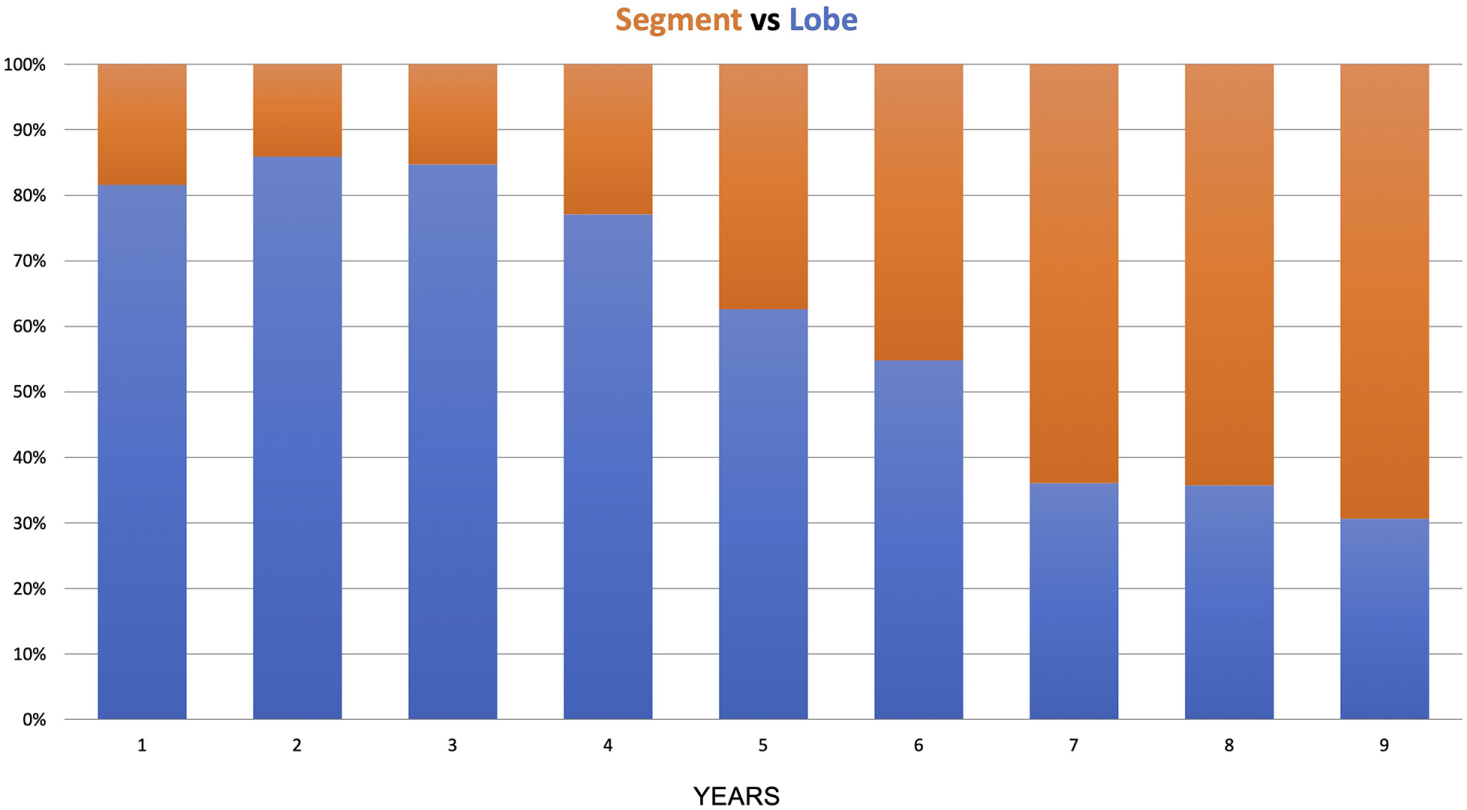
Lobe vs. segment distribution over the years.

In the anatomical lung resection group, there were 509 major resections (lobectomy, bilobectomy, pneumonectomy) and 391 segmentectomies. During the 9 years of the study, there was a shift from lobectomy to segmentectomy, with the lobectomy-to-segmentectomy ratio changing from 80%/20% in the first year to 30%/70% in the last year (Figure 4).

The population and procedure characteristics are reported in Table 3. The results were compared between the two groups. Patient characteristics were comparable between the two groups, except for a higher proportion of patients with primary lung cancer in the lobectomy group and previous cancer in the segmentectomy group. The average of staplers used per procedure did not differ between the two groups. Procedure lengths, conversion rate, and length of stay were shorter in the segmentectomy group compared to lobectomy, with a lower complication rate and equivalent Clavien–Dindo staging.

**Table 3:** Patient and procedure characteristics for anatomical lung resections.

	Lobe	Segment	*p*
*n*	509	391	
Sex M/W (%)	58/42	52/48	0.36
Mean age (years)	66.2 ± 11.9	66.7 ± 11.8	0.48
Smoking history	80 (406)	75 (293)	0.5
Previous cancer, % (*n*)	26 (134)	48 (187)	**<0** **.** **001**
Primary lung cancer, % (*n*)	79 (406)	61 (238)	**0** **.** **01**
BMI mean	25 ± 5	25 ± 5	0.8
FEV % (± ET)	88 (21)	90 (23)	0.22
Docking mean time (min)	12.4 ± 4	10.8 ± 3	**<0** **.** **001**
Console time mean time (min)	116 ± 38	99 ± 24	**<0** **.** **001**
Blood loss mean (ml)	115	67	0.006
Conversion, *n* (%)	17 (3)	2 (0.5)	**0** **.** **004**
Emergency conversion, *n* (%)	4 (0.8)	1 (0.25)	0.3
Mean number of stapler	5 ± 2.2 (382)	5.7 ± 2 (466)	0.9
LOS mean days	9.2 ± 7	7 ± 5.5	**<0** **.** **001**
Complication, *n* (%)	202 (40)	109 (28)	**0** **.** **009**
Clavien–Dindo, *n* (%)			0.45
II	144 (28)	72 (18)	
III	13 (3)	11 (3)	
IV	10 (2)	4 (1)	
V	8 (2)	2 (0.5)	

LOS, length of stay.

Bold indicates values that are statistically significant.

The primary lung cancer tumor final pathology was comparable between the two groups despite a tendency toward lower stages in the segmentectomy group, as shown in Table 4.

**Table 4:** Pathology stage for primary lung cancer*.*

	Lobe	Segment	
	406 *n* (%)	238 *n* (%)	
pT0	11 (3)	4 (2)	
pT1	232 (57)	208 (87)	
pT2	206 (26)	16 (7)	
pT3	41 (10)	8 (3)	
pT4	16 (4)	2 (1)	
			
pN0	312 (75)	224 (94)	
pN1	30 (7)	3 (13)	
pN2	73 (18)	12 (5)	
Mean number of nodes	17 ± 9	18 ± 9	*p* = 0.15

## Discussion

The results presented in this study represent the largest European monocentric prospective cohort of robotic thoracic surgery, including 1,067 consecutive procedures. In this series, 90% of the surgeries the robotic platform was used for were lung surgeries.

During this period, some modifications occurred, due to improvement of the practice over time (learning curve process) or changes in surgical policy. However, the constant was to keep the surgical technique as close as possible to what can be done during the open approach. The surgical technique employed in this study emphasizes a staple-sparing approach that focuses on manual ligation of the vessels. In 2014, there was no robotic stapler available and it was difficult to delegate vascular stapling through the port access to the nurse assistant. However, it was also useful for surgical hard skills development regarding one’s capacity to use the telemanipulator.

Therefore, this strategy was chosen to maintain surgeon proficiency and replicate open surgery techniques within a closed chest environment, favoring the development of safe sub-lobar resections. This is highlighted by the low emergency conversion rates in both groups: 0.8% for lobectomy and 0.25% for segmentectomy. Control of distal vessels in sensitive areas that need preservation can be challenging with traditional staplers or robotic staplers due to space constraints, target size mismatches, or unfavorable stapler angles.

Therefore, the average consumption of staplers per procedure did not differ significantly between the lobectomy and segmentectomy groups and remained quite low (approximately five per procedure). When stapling is used in a parenchymal plan, the use of 60 mm loads is almost twice as efficient as 45 mm ones. In addition to the technical and safety points, this also has an impact on procedure costs, as robotic staplers are expensive.

Amongst the patients who underwent anatomical lung resection, over 75% were treated for cancer (67% for primary lung cancer, 10% for metastasis disease), emphasizing the importance of radical node harvest. In the segmentectomy group, we found more early stages and also more metastasis surgery. Despite the fact that we still have limited evidence of the benefit of anatomical resection and node harvest for metastasis disease, it is still suggested by literature (15). Shiono et al. reported less local recurrence for segmentectomy vs. wedge in colorectal metastasis surgery (16), which is consistent with our approach. Despite more advanced stages and neoadjuvant chemotherapy or immuno-chemotherapy in a portion of the patients, there was no increase in morbidity, highlighting the robotic approach's suitability for advanced-stage surgery. While the conversion rate was low in both groups, it was even lower in the segmentectomy than in the lobectomy group (0.5% vs. 3%, *p* = 0.004) and only one patient had an emergency conversion in the segmentectomy group vs. four in the lobectomy group. These results indicate the safety of the surgical approach, the operating room organization, and the impact of expertise when the segmentectomy pace rose. The rates we described were lower than those first published in a single-surgeon series by Nasir et al. (9), possibly due to the use of the last generation of the da Vinci system for most segmentectomies and the vessel sewing technique we practice. Moreover, intersegmental plan definition analysis and preoperative imaging may also contribute to the end of the old paradigm, showing that tumor palpation may not be compatible with closed chest surgery.

Other complication rates were similar to that previously described in the other series (10) and were not significantly different between the groups, despite a tendency to be lower in the segmentectomy group. Notably, we observed a difference between the Si and Xi periods, which may also be influenced by a learning curve.

In our prospective cohort, the 30-day mortality was 1.6% in the lobectomy group vs. 0.5% in the segmentectomy group, which did not reach a statistical difference. These results are comparable to that of CALBG/Aliance14073 perioperative results of Altorki et al. among 697 patients with T1 less than 2 cm N0 lung cancer (17). In our study, we had a wider range of stages with patients treated from stage I to IIIB (18) with approximately 10% of patients receiving neoadjuvant chemotherapy or immuno-chemotherapy (7.4% and 4% vs. 6.3% and 3% for the lobectomy vs. segmentectomy groups, respectively). It was interesting to observe no morbidity rise despite these conditions. This highlights the importance of the robotic approach for advanced-stage surgery (19). We still have only a few publications on robotic lung surgery with significant cohorts and we call for further large-cohort studies, which is of significant importance in the era of immunotherapy.

Up to now, Epithor® contains more than 400,000 procedures, of which 11,000 were robotic procedures (2.85%), performed by 440 French surgeons, of which 152 (34.5%) performed robotic procedures. The robotic approach remains in the minority in France despite continuous growth since the first French program in 2012.

Despite the fact that this series is a single-surgeon monocentric prospective cohort, it represents approximately 10% of the robotic procedures of the database and thus is significant.

The transition from lobectomy to segmentectomy observed in this study began in the fifth year after more than 400 procedures. In the timeline, 2018 was a year when the first paper by Altorki et al. supported segmentectomy in the early stages (17). This shift in practice may reflect the growing expertise required for segmentectomy, which is more advanced than lobectomy and demands a more substantial accumulation of experience.

There are some limitations in our paper. First, it is a monocentric cohort and the external validity of the results can be questioned. Second, during the timeline, we upgraded the robotic system, and its efficiency might have an impact on the results. Third, we only focused on early outcomes.

Recent changes in lung cancer early diagnosis, treatment strategies, and enhanced care for metastatic disease increase the need for anatomical lung resection.

Dexterity and hard skill proficiency for surgeons are mandatory for sewing-and-knotting open surgery techniques. The robotic platform is the most appropriate tool thus far for minimally invasive advanced lung surgery. Its routine use in surgical practice leads to a rise in segmentectomies over lobectomies, in a safe and reproducible way, maintaining oncologic standards.

## Data Availability

The raw data supporting the conclusions of this article will be made available by the authors, without undue reservation.
